# Essential oils as promising treatments for treating *Candida albicans* infections: research progress, mechanisms, and clinical applications

**DOI:** 10.3389/fphar.2024.1400105

**Published:** 2024-05-15

**Authors:** Gao-wei Hou, Ting Huang

**Affiliations:** Zhongkai University of Agriculture and Engineering, Guangzhou, China

**Keywords:** essential oils, *Candida* albicans, antimicrobial, gut bacteria, intestinal regulation

## Abstract

*Candida albicans*: (C. alb*icans*) is a prevalent opportunistic pathogen that can cause severe mucosal and systemic fungal infections, leading to high morbidity and mortality rates. Traditional chemical drug treatments for *C. albicans* infection have limitations, including the potential for the development of drug resistance. Essential oils, which are secondary metabolites extracted from plants, have gained significant attention due to their antibacterial activity and intestinal regulatory effects. It makes them an ideal focus for eco-friendly antifungal research. This review was aimed to comprehensively evaluate the research progress, mechanisms, and clinical application prospects of essential oils in treating *C. albicans* infections through their antibacterial and intestinal regulatory effects. We delve into how essential oils exert antibacterial effects against *C. albicans* infections through these effects and provide a comprehensive analysis of related experimental studies and clinical trials. Additionally, we offer insights into the future application prospects of essential oils in antifungal therapy, aiming to provide new ideas and methods for the development of safer and more effective antifungal drugs. Through a systematic literature review and data analysis, we hope to provide insights supporting the application of essential oils in antifungal therapy while also contributing to the research and development of natural medicines. In the face of increasingly severe fungal infections, essential oils might emerge as a potent method in our arsenal, aiding in the effective protection of human and animal health.

## Introduction

*Candida albicans* (*C*. *albicans*) is the most common and important type of *Candida*, belonging to the yeast genus. Its cells are round or oval in shape, typically ranging between 3 and 5 microns in diameter. It usually exists in the form of yeast and can form mycelium under appropriate conditions. Widely distributed in the natural environment, *C. albicans* is a common symbiotic fungus that often coexists with humans and animals as part of the normal microbial community (Moran et al., 2011). Infections caused by *C. albicans* occur when the body’s immune system is compromised, resulting in conditions such as oral candidiasis (Millsop and Fazel, 2016; Vila et al., 2020), vaginal candidiasis (Gonçalves et al., 2016; Rodríguez-Cerdeira et al., 2019), skin and nail candidiasis (Jautová et al., 2001; Espinosa-Hernández et al., 2020), and severe systemic fungal infections (Blanco and Garcia, 2008). Factors contributing to infection include a compromised immune system (e.g., HIV/AIDS, organ transplantation, chemotherapy), antibiotic usage, diabetes, pregnancy, and extended catheter or ventilator use. Oral candidiasis is a prevalent *C. albicans* infection, especially among young individuals and animals with weakened immune systems. It presents as white patches or spots on the oral mucosa and tongue, accompanied by symptoms like bad breath, loss of appetite, and difficulty eating. Vaginal candidiasis can affect the genitourinary system of animals, leading to symptoms such as urinary tract inflammation, frequent urination, urgency to urinate, hematuria, vaginal inflammation, and mastitis. Symptoms of skin and nail candidiasis may include redness, swelling, itching, desquamation, and ulcers at the affected site, causing discomfort and itching in animals. In some cases, *C. albicans* infections can progress to blood infections, resulting in severe symptoms like fever, weakness, loss of appetite, anemia, and organ failure, thereby posing a serious threat to the animal’s life and health (Miceli et al., 2011).

The main antifungal drugs used for treating infections of *C. albicans* are as follows: (1) Azole antifungal drugs (Polyazoles): Azole drugs serve as the initial choice for treating the infections caused by *C. albicans*. Common polyazole drugs such as Fluconazole, Itraconazole, and Posaconazole inhibit growth and replication by disrupting yeast ketones synthesized by the cell wall of *C. albicans*; (2) Polyene antifungal drugs (Polyketides): Polyenes target the cell membrane of *C. albicans*, leading to membrane rupture and cell death. Widely used polyene drugs include Neomycin B (Amphotericin B) and the liposomal form Amphotericin B lipid complex (ABLC), typically employed in severe infections of *C. albicans*; (3) Novel antifungal drugs: In recent years, several novel antifungal agents have emerged to address refractory or drug-resistant infections of *C. albicans*, including Isaviconazole and Voriconazole, offering a wider spectrum of antifungal activities suitable for complex infections of *C. albicans* (Kathiravan et al., 2012; Cowen et al., 2015; Houšť et al., 2020).

However, the use of antibiotics pose a number of challenges. The improper and overuse of antibiotics has resulted in an escalation of bacterial resistance to these drugs. Consequently, bacteria that could typically be effectively treated with antibiotics are becoming less sensitive to the drugs, leading to infections that are difficult to cure (Balkis et al., 2002). Antibiotics not only eliminate pathogenic bacteria but also disrupt the natural microbial community within the human body. This disruption can lead to an imbalance in the gut microbiota, heightening the risk of other infections, and may impact the immune system and overall health. Residues of antibiotics enter the environment, including soil and water bodies, exerting adverse effects on ecosystems. These residues may interact with environmental bacteria, fostering the development of drug resistance in bacteria, and potentially causing harmful effects on aquatic organisms and other members of the ecosystem. Therefore, the new environmentally friendly methods which can either substitute or decrease antibiotic usage are necessary.

Essential oil is a secondary metabolite usually extracted from flowers, leaves, roots, fruits, or bark of plants through distillation, cold pressing, or solvent extraction (Valdivieso-Ugarte et al., 2019; Spisni et al., 2020). Based on a strong aroma and active ingredients (Mancianti and Ebani, 2020), it is featured by antibacterial properties that can inhibit or eradicate various microorganisms, including bacteria, fungi, and viruses (Bakkali et al., 2008). The antibacterial efficacy of different essential oils varies, stemming from their unique chemical compositions. Numerous essential oils contain volatile compounds such as phenols, alcohols, esters, aldehydes, and ketones (Dhifi et al., 2016) capable of inhibiting or eradicating microorganisms. In addition, essential oil, as a natural product, is characterized by its safety, efficiency, absence of residue, lack of resistance, good tolerance within the animal body, and minimal toxic side effects, rendering it one of the most promising antibiotic alternatives. Unlike some chemical medications, the antibacterial activity of essential oils is the result of multiple components working together. This complex combination of ingredients makes it difficult for microbes to develop microbial resistance against essential oils, thereby reducing this risk. Beyond their antibacterial effects, some essential oils also offer gut-regulating effects. They aid in balancing intestinal flora, promoting the growth of beneficial bacteria, suppressing harmful bacterial proliferation, and thereby maintaining intestinal health. These attributes hold significant implications for the prevention and treatment of infections and diseases associated with the dysregulation of gut flora.

### Antibacterial effect of essential oils

Essential oils typically consist of a variety of aldehydes, phenols, alcohols, and other chemical molecules. They are divided into two categories of compounds: terpenes (such as carvacrol and thymol) and phenylallens (such as cinnamaldehyde and eugenol), with terpenes being the predominant. Essential oil is hydrophobic and can penetrate the cell membrane of Gram-positive bacteria to enter the cell’s interior, exerting their antibacterial effect by disrupting enzyme production and protein denaturation (Pandey et al., 2017). Lipid soluble hydrophobic groups, such as hydroxyl and carbonyl groups in the structure of aldehyde phenols in essential oils, can interact with proteins within bacterial cell membranes, leading to structural and functional alterations in the membranes, membrane expansion and increased permeability, thereby eliciting antibacterial effects (Omonijo et al., 2018). The primary phenolic elements in essential oils, carvacrol, and thymol, which are isomers, are pivotal in modulating the balance of intestinal flora by changing the permeability of cell membrane and inhibiting the secretion of bacterial endotoxin.

Essential oils have antifungal properties and can inhibit or kill *C. albicans*. Ebani et al. studied the antibacterial activity of *Litsea cubeba* (Lour.) Pers. Essential oil, *Origanum vulgare* L. subsp. *Hirtum* essential oil, *Origanum majorana* L. essential oil, *Thymus vulgaris* L. essential oil, and their mixtures against pathogenic bacteria and *C. albicans*. They found that essential oil had different degrees of growth inhibition on the tested strains and *C. albicans* (Ebani et al., 2016). Hammer et al. found that concentrations of 0.25%–1.0% (v/v) of *Melaleuca alternifolia* (Maiden and Betche) Cheel oil and its components changed the permeability and fluidity of *C. albicans* (Hammer et al., 2004). Through a study on the antibacterial activity of *M. alternifolia* (Maiden and Betche) Cheel essential oil on 81 strains of *C. albicans*, it was found that the minimum inhibitory concentration for 90% of *C. albicans* was 0.25% (v/v) (Hammer et al., 1998). D'Auria et al. used *Lavandula angustifolia* Mill essential oil and its main components to inhibit *C. albicans* and found that the essential oil and its main components could inhibit the formation of bud tubes and mycelium extension of *C. albicans*, and had antibacterial and bactericidal activities as well (D’auria et al., 2005). Behmanesh et al. studied the inhibitory effect of *L. angustifolia* Mill essential oil on *C. albicans in vitro* and found that after 48 h of cultivation with *L. angustifolia* Mill essential oil, the number of fungal cells was low, indicating its antifungal effects (Behmanesh et al., 2015). Pinto et al. studied the components of *Syzygium aromaticum* (L.) Merr. and L.M.Perry essential oil and its antifungal activity and found that the essential oil and its main component eugenol could inhibit the formation of *C. albicans* bud tubes and had good antifungal activity (Pinto et al., 2009). Choonharuangdej et al. tested the bactericidal and inhibitory effects of *Cinnamomum verum* J. Presl essential oil and *Cymbopogon citratus* (DC.) Stapf essential oil on *C. albicans in vitro* and found that both essential oils could inhibit the formation of fungal biofilms (Choonharuangdej et al., 2021). Mat-Rani et al. studied the bactericidal effect of *C. citratus* (DC.) Stapf essential oil on *C. albicans* biofilm and found that 5% (v/v) essential oil could clear about 95% of the *C. albicans* biofilm, while 2.5% (v/v) essential oil had a better bactericidal effect on biofilm than a 20% (v/v) nystatin suspension. This indicates that *C. citratus* (DC.) Stapf essential oil has an obvious antifungal effect (Mat-Rani et al., 2021). Almeida et al. studied the effects of *Cymbopogon winterianus* Jowitt ex Bor and *C. verum* J. Presl essential oils on *C. albicans* biofilm and found that the minimum inhibitory concentration (MIC) of the two essential oils on *C. albicans* was 65 μg/mL and 250 μg/mL, respectively. Both oils significantly reduced the number of live bacteria and the biofilm area (Almeida et al., 2016). Banu et al. found that *C. verum* J. Presl essential oil destroyed the exopolysaccharide layer of *Candida* strains and inhibited the virulence of *C. albicans* (Banu et al., 2018). Filipowicz et al. studied the antifungal activity of *Juniperus communis* L. essential oil and found that it contained the highest concentration of (−)-α-pinene, p-cymene, and β-pinene, which had good antifungal properties. The MIC against *C. albicans* was 0.3 μg/mL (Filipowicz et al., 2003). Manoharan et al. studied the antibacterial activity of *Cedrus deodara* (Roxb. ex D. Don) G. Don essential oil on *C. albicans* and found that a 0.01% concentration of essential oil could reduce the biofilm formation of *C. albicans* by 87%. A 0.1% concentration of essential oil could completely stop the biofilm formation, indicating that *C. deodara* (Roxb. ex D. Don) G. Don essential oil has significant anti-*C. albicans* biofilm activity (Manoharan et al., 2017). Mahboubi et al. studied the chemical composition and antibacterial activity of *Mentha piperita* L. essential oil and found that it has a bactericidal effect on *C. albicans* (MIC = MLC = 0.125 μL/mL) (Mahboubi and Kazempour, 2014).

Essential oils usually contain dozens or even hundreds of chemical components. Their antibacterial activity is closely related to their chemical composition, especially some highly active chemical components. The antibacterial activity of essential oils primarily depends on their chemical functional groups. In 1996, Charai et al. reported that the activity of functional groups in plant essential oils was as follows: phenols (with the highest activity) > alcohols > aldehydes > ketones > esters > hydrocarbons (Charai et al., 1996). In 2003, Kalemba et al. summarized the antibacterial activity of functional groups in plant essential oils based on hundreds of previous studies as follows: phenols > cinnamaldehyde > alcohols > aldehydes = ketones > esters > hydrocarbons (Kalemba and Kunicka, 2003). Additionally, the same plant essential oils may show varying antibacterial and antifungal activities, suggesting that their diverse chemical components present in these oils contribute to different antibacterial and antifungal mechanisms. Table 1 presents essential oils against *C. albicans*, along with their main components.

**TABLE 1:** Essential oils against *C. albicans* along with their main components.

Essential oil	Main active components	Species	Method	Result	References
*Machilus cicatricosa* S.K. Lee	α-pinene (20.6%), bicyclogermacrene (15.2%), linalool (14.9%) and β-pinene (8.4%)	*Candida albicans* ATCC 10231	microdilution assay	MIC 32.0 μg/mL	Huong et al. (2024)
*Zingiber densissimum* rhizome essential oil	β-pinene (38.36%), β-phellandrene (26.85%) and α-pinene (13.31%)	*Candida albicans*		MIC 2 μg/mL	Nguyen-Ngoc et al. (2024)
EO extracted from the discarded peels of *Citrus depressa* Hayata	(*R*)-(+)-limonene (38.97%), *γ*-terpinene (24.39%) and linalool (6.22%)	*Candida albicans*		CD-EO exhibited inhibitory effects on the growth of *Candida* albicans	Weng et al. (2024)
*Ocimum Basilicum* L. Essential Oil	linalool 48.62%, eugenol 5.44%, trans-a-bergamotene 5.65%, a-guaiene 5.65%	*Candida albicans* ATCC 10261	broth microdilution method	MIC 1.25%, MFC 5%	Rajali et al. (2023)
EO obtained from dried flowers of *Coridothymus capitatus* (L.) Reichenb	phenols, carvacrol (67.58%) and thymol (0.16%)	*Candida albicans* ATCC 90028*, C. albicans* ATCC 10231*, C. glabrata* ATCC 90030*;* clinical isolates of *C. albicans* 183*, C. krusei* 398*, C. glabrata* 32–09*, C. norvegensis* 112*, C. lusitaniae* 103*, C. valida* 287*, C. guilliermondii* 209*, C. parapsilosis* 198*, and C. tropicalis* 16–09	broth microdilution method	MIC 0.5–8 μg/mL	Marino et al. (2020)
*Cinnamomum verum* J.Presl Essential Oil	phenylpropanoid cinnamaldehyde (82.09%), monoterpene oxide 1,8-cineole (3.1%), sesquiterpene α-copaene (3.06%)	*Candida albicans*		MIC 62.5 μg/mL	Essid et al. (2023)
*Eryngium campestre* L. Essential Oil	alpha-pinene (16.32%), Caryophyllene (16.65%), 2-oxabicyclo [9.1.0] dodeca-3, 7-diene (14.21%)	*C. albicans* (A90029)	disk diffusion method	MIC 175 mg/L, MFC 37.5 mg/L	Khoshbakhtt et al. (2023)
*Myrtus communis* L. Essential Oil	1, 8-Cineol (23.56%), alpha-pinene (17.70%)			MIC 137.5 mg/L, MFC 28.12 mg/L	
*Salvia rosmarinus* Spenn. Essential Oil	alpha-pinene (18.72%)			MIC 150 mg/L, MFC 31.25 mg/L	
*Elattaria* *cardamomum* (L.) Maton Essential Oil		*Candida albicans* ATCC 10231 (A14), *Candida albicans* ATCC 20402 (A15), Thirteen C. albicans strains isolated from different medical services (C1, C2, C3, C4, C5, 104, 104W, 108, 109, 113, 124, 126, and 118)	agar–well diffusion method and microdilution method	MICs 0.097–0.78 mg/mL, MFCs 12.5–100 mg/mL	Noumi et al. (2022)
EOs from the seeds of *Nigella sativa* L., the peels of *Cinnamomum verum* J.Presl		*C. albicans* (ATCC 10231)	microdilution method	MIC 12.5 mg/mL, MFC 25 mg/mL	Mirzaii et al. (2021)
*Cinnamomum verum* J.Presl Essential Oil		*C. albicans* ATCC 10261	broth microdilution method	MIC 93.75 μg/mL	Tomičić et al. (2022)
Chinese herbal plant *Pogostemon cablin* (Blanco) Benth	*Patchouli alcohol*	*C. albicans* SC5314 and ATCC 10231, *C. glabrata* ATCC 2001, *C. parapsilosis* ATCC22019, *C. krusei* ATCC6258, *C. tropicalis* ATCC7349		MICs 64 μg/mL, MFCs 64–128 μg/mL	Zhang et al. (2024)
*Origanum majorana* L. Essential Oil	carvacrol (75.3% and 84%)	*C. albicans* ATCC 90028, *C. albicans* MFBF 10778, *C. albicans* MFBF 11100, *C. tropicalis* ATCC 750, *C. krusei* ATCC 14243, *C. dubliniensis* MFBF 11098	Microdilution Assay	IC_50_ < 0.0156–0.25 μg/mL, IC_90_ 0.5 μg/mL	Kaskatepe et al. (2022)
*Cinnamomum verum* J.Presl essential oil		*C. albicans* ATCC 10231	Kirby–Bauer method (disk diffusion)	100% EOC 24-h group 22.1 ± 11 mm, 48-h group 31.2 ± 3.2 mm	Hurtado et al. (2020)
*Cymbopogon citratus* (DC.) Stapf Essential oil	geranial (48.2%), neral (37.49%)	*Candida tropicalis*, *Candida catenulate*, *Candida albicans*, *Candida parapsilosis* ATCC 22019, *Candida krusei* ATCC 6258	broth microdilution method	MIC 1.25–5 μL/mL	Rhimi et al. (2022)
*Cymbopogon Proximus* Essential oil	piperitone (66.99%), α-terpinolene (15.7%)			MIC 2.5–20 μL/mL	
*Cymbopogon flexuosus* (Nees ex Steud.) Will.Watson Essential oil	29.4% geranial (transcitral, a-citral) and 30.4% neral (cis-citral, b-citral)	*C. albicans* SC5314, *C. tropicalis* ATCC1369	serial microdilutions	MIC 0.0781%, 0.039%	Gao et al. (2020)
*Atriplex halimus* L. Essential oil	viridiflorol (40.23%), phytol (18.24%), germacrene D (6.94%)	*Candida albicans*	Disc Diffusion Method	resistant	Soltani et al. (2023)
*Centaurium erythraea* Rafn Essential oil	β-copaen-4α-ol (38.41%), manool (8.2%), carvacrol (6.43%)			inhibition diameter of 18.5 ± 3.53 mm	
*P. amboinicus* Essential oil	thymol (0.13%–0.16%), carvacrol (68.92%–75.21%)	*Candida rugosa* (IZ-12), *C. kruseii* (ATCC 6258), *C. tropicalis* (CBS 94), *C. dubliniensis* (CBS7987), *C. albicans* (ATCC 90028), *C. utilis* (CBS 5609), *C. kruseii* (CBS 572), *C. lusitanea* (IZ-06), *C. gablata* (IZ-07), *C. gablata* (ATCC 5207), *C. albicans* (CBS 562)	disc-diffusion technique	C. albicans ATCC 90028 and C. dubliniensis inhibition zone (42.0364 ± 0.0023 and 40.0553 ± 0.0049 mm), C. kruseii (28.0125 ± 0.0007 mm), The other selected *Candida* spp. 31.0976 ± 0.0051–38.0905 ± 0.0031 mm	Mendonça et al. (2023)
*Origanum vulgare* L. essential oil	cis-sabinene hydrate (16.72%), 4-terpineol (13.57%), thymol (14.20%) and γ-terpinene (11.66%)	*C. albicans* ATCC 90029, *C. albicans* ATCC 10231, *C. krusei* ATCC 6258	broth microdilution assay	MIC 0.01 μg/mL, 0.97 μg/mL and 5.33 μg/mL	Cid-Chevecich et al. (2022)
Essential Oil from the Leaves of *Tapirira guianensis Aubl*	eugenol (59.00%), α-copaene (0.40%), β-caryophyllene (29.91%), α-humulene	*Candida albicans* fungal strains 0131, 0128, 0102, and 0104	broth microdilution MIC tests	MIC 156–312 μg/mL and MFC 312–625 μg/mL	Oliveira et al. (2024)
*Mentha suaveolens* Ehrh. Essential Oils	PO, piperitenone (PIP), nepetalactone (NPL), p-cymen-8-ol (PCY), limonene (LIM), and cis-piperitone epoxide (CPO)	*Candida albicans* (ATCC 10231)	microbroth dilution method (microsterile plate)	MIC 0.39–0.78 mg/mL (0.039% and 0.078% p/v)	Tadić et al. (2023)
*Ferula macrecolea* essential oil	terpinolene (71.25%), n-nonanal (6.32%), and linalool (3.95%)	*C. albicans* ATCC 5027, *C. albicans* ATCC 76616	broth-microdilution approach	MIC and MFC on C. albicans sensitive to nystatin:1.6 and 2.0 μg/mL. MIC and MFC on nystatinresistant strains:3.3 and 4 μg/mL	Sadeghi et al. (2023)
*Rosmarinus officinalis* Spenn. Essential Oils	1,8-cineole (4.81%–37.83%), α-pinene (13.07%–51.36%), and camphor (11.95%–24.30%)	*Candida albicans* (ATCC 90028)	broth microdilution method	MICs 0.781, 0.781, and 1.56 μg/mL	Al-Maharik et al. (2022)
*Cinnamomum verum* J.Presl *Leaf and Syzygium aromaticum* (L.) Merr. and L.M.Perry Essential Oils	eugenol (>70%)	*C. albicans* RSY150, *C. albicans* clinical strains (ATCC 10231 clinical reference, blood, genital, and fluconazole resistant isolates)	broth microdilution method	MICs of 600 and 500 μg/mL against RSY150 and 1000 and 750 μg/mL for ATCC 10231. Combined oils are additive (FICI 0.72 ± 0.16) and synergistic (0.5) against RSY150 and the clinical reference strain	Shahina et al. (2022)
*Cinnamomum verum* J.Presl leaf essential oil	eugenol (77.22%)	*C. albicans* (ATCC MYA-2876), *C. tropicalis* (ATCC 750) and *C. dubliniensis* (ATCC MYA-646)	broth microdilution	MIC 1.0 mg/mL	Wijesinghe et al. (2020)
*Zataria Multiflora* Boiss, *Mentha Longifolia* (L.) L., *and Origanum Vulgare* L. Plant Essential Oils		*Candida albicans*	micro dilution method	the highest resistance rates in 0.625 mg/mLof *O. vulgare*, *M. longifolia*, and *Z. multiflora* EOs were 31(77.5%), 15(37.5%), and 13 (32.5%), the lowest MIC of *Z. Multiflora* EO was 0.625 mg/mL	Hossrini et al. (2022)
*Syzygium aromaticum* (L.) Merr. and L.M.Perry essential oil	eugenol (84.64%), β-caryophyllene (12.76%)	*C. albicans* (ATCC 10231) and 15 clinical *C. albicans* isolates	broth microdilution technique	MIC 625–1250 μg/mL	Hekmatpanah et al. (2022)
*Cinnamomum verum* J.Presl leaf essential oil	eugenol (77.22%)	*C. albicans* (ATCC MYA2876), *C. tropicalis* (ATCC 750), *C. dubliniensis* (ATCC MYA-646)	CLSI microdilution assay	MIC 1.0 mg/mL, MFC 2.0 mg/mL	Wijesinghe et al. (2021)
*Grapefruit Peels* Essential Oil	DL-limonene (79.85%), by β-myrcene (3.13%) and noontkatone (2.04%)	*Candida albicans* ATCC (10,231)	2,5-diphenyl-2Htetrazolium bromide (MTT) assay	EO was observed to be toxic to pathogenic C. albicans at aconcentration range of 3200–400 μg/mL at 8h	Yaldiz et al. (2022)
EOs from *Pentadiplandra brazzeana Baillon (PB)* root and *Drypetes gossweileri S. Moore (DG)* stem bark		*Candida albicans* ATCC P37037 and *Candida parapsilopsis* ATCC 22,019	broth microdilution technique	MICs of D. gossweileri EO were obtained at 62.50 μg/mL for C. albicans and 125 μg/mL for C. parapsilopsis. MICs of P. brazzeana EO were 62.50 μg/mL for C. albicans and 250 μg/mL for C. parapsilopsis	Tchinang et al. (2023)
*Cinnamomum verum* J.Presl bark and leaf essential oils	CLEO: eugenol (62.57%)	*C. albicans* ATCC 10231, *C. albicans* ATCC 2091, *C. auris:* NCPF 8971	disc diffusion (direct and vapour) and broth microdilution method	MICs and MFCs of bark CEO for all tested strains were below 0.03% (v/v), MICs of leaf CEO were 0.06%–0.13% (v/v) and MFCs at 0.25% (v/v)	Tran et al. (2020)
	CBEO: trans-cinnamaldehyde (66.43%)				

### Intestinal regulation of essential oils

The normal gut microbiota typically can suppress the proliferation of *C. albicans*. However, when the body’s immune system is compromised, antibiotics are used long-term, a poor diet is followed, or other diseases are present, the balance of gut microbiota may be disrupted. This disruption can result in an overgrowth of *C. albicans*, leading to conditions like *Candida* enteritis and related diseases. Several associations between *C. albicans* overgrowth and gut health issues include: (1) *Candida* Enteritis: This condition, marked by symptoms such as abdominal pain, diarrhea, indigestion, loss of appetite, weight loss, and inflammation, can negatively impact gut health (Rusu et al., 2020); (2) Leaky gut: Overgrowth of *C. albicans* may trigger “leaky gut”, characterized by damage and increased permeability of the intestinal mucosa, allowing harmful substances such as bacteria and toxins to enter the bloodstream, causing inflammation, immune system dysfunctions, and other health complications (Panpetch et al., 2020); (3) Effects on immune system: The overgrowth of *C. albicans* might disrupt immune system functions, interfering with immune cell activities and responses, subsequently elevating the risk of infections. Additionally, excessive growth of *C. albicans* can produce toxins that adversely affect the immune system (Tong and Tang, 2017); (4) Nutrient absorption problems: The overgrowth of *C. albicans* can impede the normal absorption of the intestine, affecting the nutritional status and overall health of the host (Martins et al., 2014).

*Candida albicans* infection may lead to an imbalance in the gut microbiota, which refers to a change in the proportion of beneficial and harmful bacteria in the microbiota. Under normal circumstances, the intestinal flora maintains a state of balance, with beneficial bacteria such as *Lactobacillus* and *Bifidobacterium*, inhibiting the growth of harmful bacteria, supporting the health of the intestinal mucosa, and providing nutritional support. However, *C. albicans* infection may result in an increase in harmful bacteria and a decrease in beneficial bacteria, potentially causing an imbalance in the flora. Given that *C. albicans* infection is a fungal infection, the number of fungi in the gut may increase significantly during infection, negatively impacting the diversity of flora, which signifies the richness and balance of various microorganisms in the flora (Cheng et al., 2023). Infection can cause alterations in the flora and an increase in dominant bacteria, ultimately lowering overall diversity (Wang et al., 2021). Furthermore, *C. albicans* infection may alter bacterial attachment and adhesion. The fungus can attach to the intestinal mucosa and form biofilms, creating a conducive environment for its growth and reproduction. This process may interfere with the ability of other bacteria to attach and adhere to the intestinal tract. The infection might also influence the intestinal immune system, potentially leading to an abnormal immune response triggering the activation of immune cells and an increase in inflammatory responses, further disrupting the balance of intestinal flora (Zeise et al., 2021). The impacts of *C. albicans* infection on intestinal flora are shown in Table 2.

**TABLE 2:** The impacts of *C. albicans* infection on intestinal flora.

Species	Animals	Changes in the composition of microbiota flora	Diversity of microbiota	Inflammatory response	Regulation of immunity	Intestinal mucosal barrier	Reference
*C. albicans* (strain SC5314)	female C57BL/6 mice	Probiotics (Lactobacillus, butyrateproducing bacteria, including Roseburia, Lachnospiraceae, and Clostridia) were depleted; pathogenic	the intestinal flora abundance was reduced			exacerbates changes in colon histopathology and increases intestinal permeability; the reduced expression of the intestinal tight junction proteins ZO-1, ZO-2, Claudin-1, and Occludin in the model group also proved the damage to the intestinal integrity	(Hu et al., 2021)
		Bacteria (Escherichia-Shigella and Proteus, belonging to the Proteobacteria phylum, and the inflammation mediators Ruminococcus and Parabacteroides) were enriched					
*C albicans* ATCC90028	8-week-old male C57BL/6 mice	enhance the fermentation of some specific fecal bacteria		increased serum IL-6 and TNF-α (pro-inflammation) and decreased serum IL-10 (anti-inflammation)			(Panpetch et al., 2020)
*C. albicans* SC5314 strain	male and female C57BL/6 J mice		lead to an increased relative abundance of Proteobacteria (75%) and Cyanobacteria (20%) members, higher relative abundance in Verrucomicrobiaceae, Rickettsiales and Proteobacteria members were observed with C. albicans infection				(Gutierrez et al., 2020)
*Candida albicans* ATCC 90028	Male 8-week-old C57BL/6 mice		increased Bacteroides with decreased Firmicutes and reduced bacterial diversity			enhanced the worsening of gut leakage and microbiota alterations	(Panpetch et al., 2021)
*C. albicans* strain SC5314	female C57BL/6 (B6) mice and immunocompromised randomized male and female Rag2−/−IL2γc−/− (Rag2γc) mice			a significant increase in circulating proinflammatory cytokines, including IL-6, TNF-α, and IL-10, lower levels of IL-22			(Pan et al., 2021)
*C. albicans* SC5314, *C. albicans* strain 529L, *C. albicans* tup1Δ/Δ homozygous deletion mutant	Six to nine-week-old female C57BL/6 mice	the most dominant taxa were Stenotrophomonas, Alphaproteobacteria and to a lesser extent Enterococcus in the jejunum	increase in bacterial diversity in the jejunum				(Bertolini et al., 2019)
*C. albicans* (SC5314 strain)	C3H/HeN mice (6 to 8 weeks)	cause the reduction of Ralstonia, Alistipes, Clostridia UCG-014, Ruminococcus, and Lachnospiraceae NK4A136 group	induce the decrease of alpha diversity of bacteria and fungi in the gut microbiome		γδT cell neutralisation boosted the overgrowth of C. albicans. Additionally, IL-17A neutralisation aggravated the microbial dysbiosis of bacteria and fungi caused by C. albicans infection		(Wang et al., 2023)
*C. albicans* (strain SC5314)	Female C57BL/6 mice aged 8 weeks					proteins Claudin-1 and Occludin in the colon were significantly depleted	(Hu et al., 2022)
*C. albicans*	wild-type NMRI mice		stably cohabited with individual bacterial commensals, such as B. thetaiotaomicron and L. reuteri,in the murine intestine				(Eckstein et al., 2020)
*Candida albicans* (strain SC5314)	Six to eight week-old male and female C57BL/6 mice			the mucosal layer was with inflammatory cell infiltration		ulcers in the superficial layer of the colonic mucosa, entire tissue structure of the mucosal layer was destroyed	(Wang et al., 2022)
*Candida albicans* wild-type SC5314 and its yeast-restricted mutant HLC54 (cph1/cph1 efg1/efg1)	C57BL/6 female mice, 6- to 8week-old Rag2-/-IL2gc-/- (Rag2gc) male and female mice	a positive correlation between the B. vulgatus abundance and fungal load was found, and the negative correlation between the Candidatus Arthromitus abundance	significant increase in the abundance of Firmicutes, particularly Lachnospiraceae and Ruminococcaceae, as well as a significant decrease in the abundance of Candidatus Arthromitus			affect the differentiation of intestinal Th17 cells	(Yan et al., 2024)
*C. albicans* strains SC5314	Female C57BL/6 mice (6 to 8 weeks old)		the Proteobacteria were enriched while the other phyla were markedly reduced	serum IL-6, IL-10, TNF-α were significantly higher	IL-17A, IFN-γ, IL-12, and IL-22 were significantly decreased		(Li et al., 2020b)
*C. albicans* strain CHN1	Female C57BL/6 mice	predominantly Lactobacillus spp	changes the diversity of the cecal bacterial populations				(Mason et al., 2012)

Intestinal flora refers to the microbial community in the human digestive system, playing a critical role in human health and immune function. Imbalances in intestinal flora are associated with the occurrence and development of various diseases. Essential oils are believed to contain diverse active ingredients that could potentially influence gut microbiota and health, but further research is necessary to understand these mechanisms. The compounds found in essential oils can act as prebiotics, providing nutrients and a growth-friendly environment for beneficial bacteria. These ingredients can be used by beneficial bacteria in the gut to promote their growth and reproduction. For example, dietary fiber, a common prebiotic, shares similarities with certain components in essential oils such as natural polysaccharides. Kondapalli et al. conducted oral tests of *Ocimum sanctum* L., *Zingiber officinale* Roscoe, and *Piper nigrum* L. essential oil on healthy rats and found that the three essential oils had high prebiotic potential and could promote the growth of beneficial intestinal bacteria (*Lactobacillus* and *Bifidobacterium*) (Kondapalli et al., 2022). Babu et al. found that *O. sanctum* L., *Z. officinale* Roscoe, and *P. nigrum* L. extracts contain high concentrations of polyphenols, which have a proliferation effect on some intestinal microbiota and have high probiotic potential (Babu et al., 2018). Phenolic compounds or polyphenols are widely existing secondary metabolites in the plant kingdom, which can maintain the balance of intestinal microorganisms by stimulating the growth of beneficial bacteria (such as *Lactobacillus* and *Bifidobacterium*) and inhibiting pathogenic bacteria, and play a prebiotic role, thus contributing to the maintenance of intestinal health (Dueñas et al., 2015). Oligosaccharides in plant extracts have a prebiotic effect and contribute to the growth of *Lactobacillus* and *Bifidobacterium* and the inhibition of *Bacteroides* (Markowiak and Śliżewska, 2017). Leong et al. studied the regulatory effects of *Pogostemon cablin* (Blanco) Benth essential oil on intestinal microbiota in mice and found that *P. cablin* (Blanco) Benth essential oil had significant prebiotic-likes effects (Leong et al., 2019). Some of the ingredients in essential oils have antibacterial activity that can inhibit the growth and reproduction of harmful gut bacteria, thereby mitigating their detrimental effects and preserving the balance of intestinal flora. For example, camellia alcohol in *M. alternifolia* (Maiden and Betche) Cheel essential oil has a broad spectrum of antibacterial activity, effectively restraining the proliferation of various harmful bacteria. Studies have demonstrated that *Foeniculum vulgare* Mill. Seed essential oil has significant inhibitory effects on *Acinetobacter baumannii*, *Escherichia coli*, and *Staphylococcus aureus* (Barrahi et al., 2020). Additionally, *O. vulgare* L. subsp. *Hirtum*, *C. verum* J. Presl, *S. aromaticum* (L.) Merr. and L.M.Perry, *Thymus vulgaris* L., and *M. alternifolia* (Maiden and Betche) Cheel essential oils have strong antibacterial effects against *Salmonella enterica* and *Listeria monocytogenes* (Mazzarrino et al., 2015). *Cymbopogon schoenanthus* (L.) Spreng. Essential oil has a good antibacterial effect on *E. coli*, *Staphylococcus aureus*, and *methicillin-sensitive staphylococcus aureus* (Hashim et al., 2017). Certain components within essential oils can regulate the acid-base balance of the intestine, influencing the microecological environment of the gut. Some beneficial bacteria thrive in acidic environments, while harmful bacteria prefer alkaline environments. By adjusting the pH of the gut, essential oils can create a favorable environment for the growth of beneficial bacteria and inhibit the proliferation of harmful bacteria. Giannenas et al. evaluated the effects of dietary supplementation of benzoic acid or thymol and its essential oil mixture on growth performance of Turkey, and found that essential oil mixture decreased the pH values of the caecal content, increased lactic acid bacteria, and decreased coliform group (Giannenas et al., 2014). Dibner et al. studied the nutrition and metabolism of organic acids on intestinal flora and found that organic acids can reduce the pH values of intestinal digesta and participate in antibacterial activity (Dibner and Buttin, 2002). Moreover, specific components in essential oils have anti-inflammatory properties that aid in promoting intestinal health by reducing inflammatory responses. *Ocimum basilicum* L. essential oil, for instance, has been shown to mitigate tissue damage and myeloperoxidase (MPO) activity caused by colitis (Rashidian et al., 2016). *Foeniculum vulgare* Mill. Essential oil has been found to reduce histological lesions from colitis and impact the expression of MPO, tumor necrosis factor α, and nuclear factor-κB mucosal mRNA levels (Rezayat et al., 2018). Furthermore, some essential oils show positive effects on the growth and activity of beneficial bacteria, leading to an increase in beneficial bacteria populations while decreasing harmful ones. This reduction in intestinal oxidative stress is crucial for maintaining intestinal health. Research indicates that the number of *Lactobacillus jejuni* increased, while the number of *Enterococcus* and *E. coli* decreased in piglets treated with a carvhol-thymol mixture (Wei et al., 2017). Similarly, the administration of carvall essential oil to broilers showed a reduction in *Salmonella* and *E. coli* populations in their intestines (Liu et al., 2018). Sahoo et al. added *Curcuma longa* L. and *Z. officinale* Roscoe to the diets of broilers, it was observed that the growth of several pathogenic bacteria in the chickens’ intestines was restricted, ultimately contributing to balanced intestinal microflora and improved feed utilization (Sahoo et al., 2019). The regulatory effects of essential oils on gut health are shown in Supplementary Table S1.

### Clinical application

The primary applications of essential oils in infections of *C. albicans* mainly involve the treatment of oral and skin infections. Here are the main applications of essential oils in these areas are as follows:

(1) Oral infection: (Ⅰ) *M. alternifolia* (Maiden and Betche) Cheel oil: *M. alternifolia* (Maiden and Betche) Cheel oil can be used as an oral mouthwash to treat oral infections of *C. albicans*. Its antifungal and antibacterial properties can reduce the growth and spread of *C. albicans*; (Ⅱ) *S. aromaticum* (L.); Merr. and L.M.Perry essential oil: *S. aromaticum* (L.) Merr. and L.M.Perry essential oil is effective in managing oral infections of *C. albicans* due to its potent antifungal and antibacterial effects.

(2) Skin infection: (Ⅰ) *M. alternifolia* (Maiden and Betche) Cheel oil: *M. alternifolia* (Maiden and Betche) Cheel oil can be topically applied to address skin infections of *C. albicans* such as *candida* dermatitis. It can be directly applied to affected areas, possessing antifungal and antibacterial effects; (Ⅱ) *L. angustifolia* Mill. Essential oil: *L. angustifolia* Mill. Essential oil is effective in treating skin infections of *C. albicans*. With its antifungal and anti-inflammatory properties, it can alleviate itching and reduce inflammatory responses; (Ⅲ) *C. verum* J. Presl essential oil: *C. verum* J. Presl essential oil can be topically used to manage skin infections of *C. albicans*. Despite its antifungal and antibacterial activity, caution should be exercised to prevent skin irritation; (Ⅳ) Other essential oils: *F. vulgare* Mill. Essential oil, *Salvia rosmarinus* Spenn. Essential oil, *Cupressus funebris* Endl. Essential oil, *C. citratus* (DC.) Stapf essential oil, and *Eucalyptus robusta* Sm. Essential oil are also beneficial for the topical treatment of skin infections of *C. albicans*. They possess antifungal and antibacterial properties that can relieve symptoms and aid in wound healing.

**FIGURE 1:**
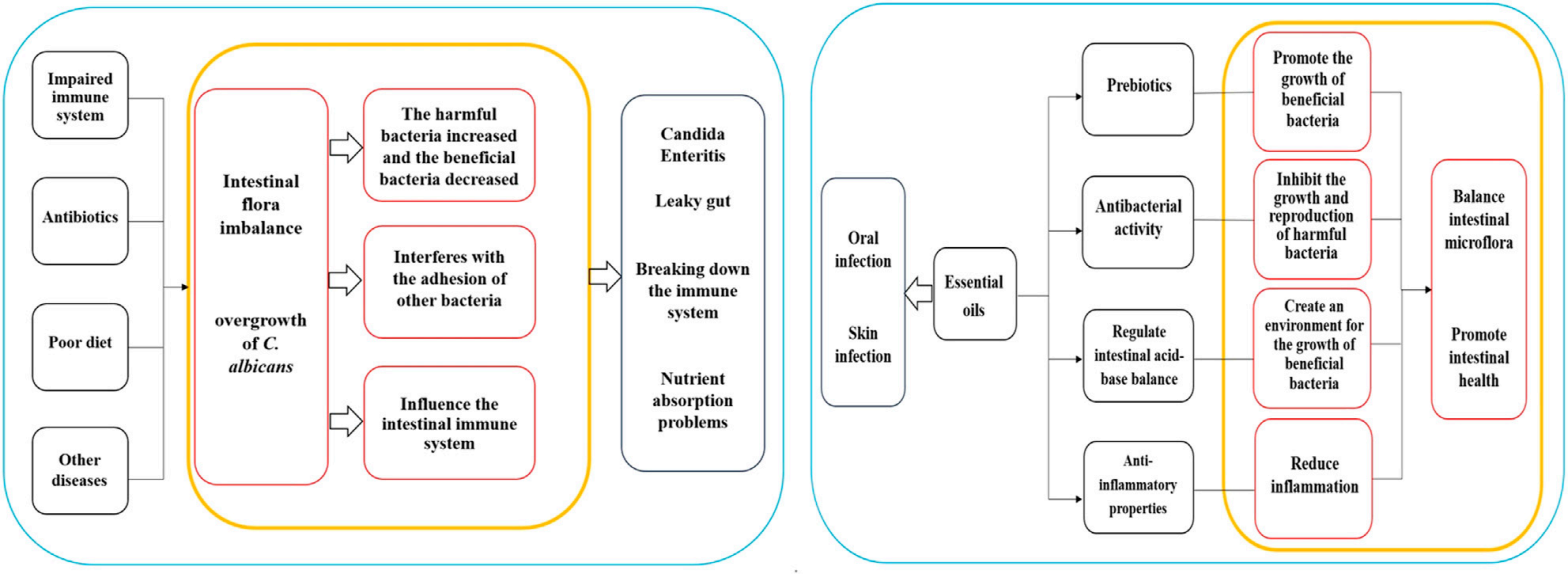
Mechanism diagrams of the Intestinal regulation of essential oils and Clinical application.

It's important to note that the application of essential oils should follow the appropriate dilution ratio and application method. In cases of oral infections, essential oils can be diluted and used as a mouthwash. Regarding skin infections, essential oils should be mixed with a carrier oil and gently applied to the affected area. It is best to perform a skin sensitivity test before usage and it is essential to adhere to the recommended guidelines for application. Although essential oils show some potential in the treatment of infections of *C. albicans*, further research is necessary to validate their effectiveness and safety. The mechanism diagrams of the Intestinal regulation of essential oils and Clinical application are shown in Figure 1.

### Challenges and prospects

As environmental regulations become more stringent, there is a growing consensus on the need to reduce and replace resistance. People are growing more health-conscious and environmentally aware, leading to a surge in interest in promoting natural and eco-friendly plant essential oils as a burgeoning research Frontier and focal point of study. Presently, numerous scholars both domestically and internationally have conducted extensive research on essential oils, focusing particularly on their physical and chemical properties, biological activities, mechanisms of action, etc. However, the majority of studies regarding the antibacterial activity of essential oils are conducted based on the MIC of these oils against bacteria or fungi. There is no systematic and thorough exploration into the antibacterial kinetics and mechanisms related to essential oils, indicating considerable groundwork to be undertaken. Specifically, understanding the mechanisms behind the antibacterial and antifungal effects of essential oils, exploring potential synergistic antibacterial properties among different oils, and investigating the relationship between chemical components require further investigation.

Essential oils represent a promising biological resource sourced from a wide variety of origins, known for their low toxicity and minimal side effects. The future industrialization of essential oils should not be confined to specific sectors such as traditional medicine, food, or daily necessities. Instead, it should incorporate a multidisciplinary, multi-directional, and versatile approach into its development. With the continuous advancement of various separation and detection technologies, the composition, structure, and functions of plant essential oils are becoming increasingly elucidated, leading to expanded applications and developmental opportunities in areas such as medicine, healthcare products, disease and pest prevention, food, and environmental protection (Rath, 2007; Pavela and Benelli, 2016).

## Conclusion

Essential oils have demonstrated their ability to combat the infections of *C. albicans* through various antibacterial mechanisms and by regulating intestinal flora. Further research and clinical trials on essential oils are imperative to comprehensively assess their efficacy and safety as a viable treatment approach for the infections caused by *C. albicans*.
