# Supplementary material for: Essential oils as promising treatments for treating Candida albicans infections: research progress, mechanisms, and clinical applications
Source: Front Pharmacol. 2024 May 15;15:1400105. doi: 10.3389/fphar.2024.1400105 (PMC11145275; doi:10.3389/fphar.2024.1400105)
Supplement: Supplementary file 1 [file Table1.docx]

**Table S1.** The regulatory effects of essential oils on gut health

| **Essential oil** | **Main components** | **Animals** | **Changes in the composition of bacterial flora** | **Changes in abundance** | **Changes in metabolism** | **Intestinal barrier** | **Intestinal immunity** | **Health** | **Reference** |
| --- | --- | --- | --- | --- | --- | --- | --- | --- | --- |
| 5 g/tonne *thymol* and 5 g/tonne *cinnamaldehyde* |  | broilers | increases in the proportions of *Lactobacillus* and *Escherichia coli* |  | Butyrate，spermine increase；acetic acid，propionic acid， isovaleric acid，tyramine decreased |  |  | improving broiler performance | (Tiihonen et al., 2010) |
| *oregano* EO at 100, 200 and 400 mg/kg (EO100, EO200 and EO400) |  | laying hens |  | increased the abundances of Burkholderiales, Actinobacteria, Bifidobacteriales, Enterococcaceae and Bacillaceae, whereas decreased Shigella abundance in the ileum. |  | quadratic elevation in ileal chymotrypsin and lipase activity, along with a linear increase in villus height to crypt depth ratio, Quadratic declines in mRNA expression of IL-1β, TNF-α, IFN-γ and TLR-4, concurrent with a linear and quadratic increase inZO-1 expression were identified in the ileum |  | resulted in higher eggshell thickness | (Feng et al., 2021) |

**Table S1.** *Cont.*

| **Essential oil** | **Main components** | **Animals** | **Changes in the composition of bacterial flora** | **Changes in abundance** | **Changes in metabolism** | **Intestinal barrier** | **Intestinal immunity** | **Health** | **Reference** |
| --- | --- | --- | --- | --- | --- | --- | --- | --- | --- |
| *cinnamon* essential oil | containing approximately 68.95% cinnamaldehyde | mice |  | improved diversity and richness of intestinal microbiota, and a modified community composition with a decrease in Helicobacter and Bacteroides and an increase in Bacteroidales_S24‐7 family and short‐chain fatty acids (SCFA)‐producing bacteria (Alloprevotella and Lachnospiraceae_NK4A136_group) |  |  |  | effectively relieved the colitis inflammation | (Li et al., 2020a) |
| 300 mg/kg diet of *rosemary* and 300 mg/kg diet of *cinnamon* essential oils |  | laying hens |  |  |  |  | immunity and antioxidant parameters were not significant | significant positive effects on hen performance and egg production | (Abo Ghanima et al., 2020) |
| *bitter* *orange* (*Citrus* *aurantium*) essential oil | linalyl acetate (42.77%) | common carp |  |  |  | Cell infiltration in the lamina propria and submucosa was found to in higher oil does |  | NO0.25 had the highest growth efficiency | (Acar et al., 2021) |

**Table S1.** *Cont.*

| **Essential oil** | **Main components** | **Animals** | **Changes in the composition of bacterial flora** | **Changes in abundance** | **Changes in metabolism** | **Intestinal barrier** | **Intestinal immunity** | **Health** | **Reference** |
| --- | --- | --- | --- | --- | --- | --- | --- | --- | --- |
| *oregano* essential oil at 300 mg/kg, *garlic* essential oil at 300 mg/kg and *oregano* essential oil at 150 mg/kg + *garlic* essential oil at 150 mg/kg |  | broiler chicks | clostridium counts were significantly decreased |  |  |  |  | no differences in feed intake and feed conversion efficiency. | (Kırkpınar et al., 2011) |
| *fennel* essential oil | Anethole (75%), Fenchone (16%), α-a pinene (4%), limonene (3.3%) | rabbits |  |  | lower total cholesterol, triglyceride, and MDA |  |  | positively affect the growth performance | (Imbabi et al., 2021) |
| *oregano* (50 g/kg premix) and *garlic* (5 g/kg premix) essential oils |  | broiler chicks | lower total anaerobe counts in the caecal digesta, higher counts of E. coli and Enterobacteriaceae, and lower counts of clostridium perfringens in the jejunum digesta, |  |  |  |  | improved final body weight and feed conversion ratio | (Sidiropoulou et al., 2020) |

**Table S1.** *Cont.*

| **Essential oil** | **Main components** | **Animals** | **Changes in the composition of bacterial flora** | **Changes in abundance** | **Changes in metabolism** | **Intestinal barrier** | **Intestinal immunity** | **Health** | **Reference** |
| --- | --- | --- | --- | --- | --- | --- | --- | --- | --- |
| plant essential oils | 78.3% Cinnamic dehyde, 4% Isophorone, and 2.7% eugenol | chickens |  | significantly increased the relative abundance of phyla Bacteroidetes and decreased the abundance of phyla firmicutes and genus of Lactobacillus in cecal microbiota, the relative abundance of genus of Alistipes, unclassified Rikenellaceae, Roseburia, and Anaeroplasma was enriched |  |  |  | altered the composition and metabolism profile of the cecal microbiota, modified the serum metabolism profile | (Chen et al., 2020) |
| *Oregano* essential oil | carvacrol and thymol | White sows and their piglets |  | increase in the relative abundance of Lactobacillaceae family (sows); relative decrease in Enterobacteriaceae and increase in butyrate producers (Lachnospiraceae family) (piglet) |  |  |  | improved average daily weight gain | (Hall et al., 2021) |
| essential oils product (50, 100 or 150 g/tonne) | thymol and cinnamaldehyde | piglets | decreased E. coli counts in feces |  |  |  |  | improve performance, immunity and gut microflora of newly weaned pigs | (Li et al., 2012b) |

**Table S1.** *Cont.*

| **Essential oil** | **Main components** | **Animals** | **Changes in the composition of bacterial flora** | **Changes in abundance** | **Changes in metabolism** | **Intestinal barrier** | **Intestinal immunity** | **Health** | **Reference** |
| --- | --- | --- | --- | --- | --- | --- | --- | --- | --- |
| *oregano* essential oil (150 or 300 mg/kg) |  | yellow-feathered chickens |  | increased the relative abundance of Firmicutes phylum, and clostridium and Lactobacillus genera, and decreasing that of Romboutsia. |  | increased the content of secretory immunoglobulin A and the relative expression of Claudin 1, Mucin 2, and Avain beta-defensin 1 in ileum |  | improved production of natural antibodies, and favorably modulated intestinal microbiota composition | (Ruan et al., 2021) |
| *lavender* essential oil (300, 600 mg/kg) |  | broiler chickens | decreased Escherichia coli population in the ileum and cecal contents |  |  | increased villus height and villus height to crypt depth of jejunum |  | improve growth performance, gut microbiota balance, intestinal morphology, and antioxidant activity | (Barbarestani et al., 2020) |
| 25.0 mg/kg of an essential oil blend from star anise, *rosemary*, *thyme* and *oregano* (essential oils) |  | broilers |  |  |  | increased the villus height and the villus/crypt ratio of the intestine |  | promoting the growth performance | (Youssef et al., 2021) |

**Table S1.** *Cont.*

| **Essential oil** | **Main components** | **Animals** | **Changes in the composition of bacterial flora** | **Changes in abundance** | **Changes in metabolism** | **Intestinal barrier** | **Intestinal immunity** | **Health** | **Reference** |
| --- | --- | --- | --- | --- | --- | --- | --- | --- | --- |
| *oregano* essential oil (500, 1500,4500 mg/kg) |  | cyprinus carpio (fish) |  | genera Propionibacterium, Brevinema, and Corynebacterium_1 were enriched in the OEO-H group |  |  |  | increased digestive enzyme activity and antioxidant capability, stimulated immunomodulatory effects, and enhanced disease resistance | (Zhang et al., 2020) |
| *ajwain* and *dill* essential oils (0.25 g/kg) |  | Japanese quail |  |  |  | the intestinal morphometric indices increased |  | positive effect on growth performance | (Hazrati et al., 2020) |
| essential oil (30 mg/kg) |  | piglets |  |  |  | increased villous height of duodenum |  | improved performance | (Xu et al., 2018) |
| essential oils | cinnamaldehyde (15%), thymol (5%) | piglets |  | higher relative abundance of Lactobacillus mucosae |  |  |  | improved the growth performance and modulated the microflora community | (Yang et al., 2019) |

**Table S1.** *Cont.*

| **Essential oil** | **Main components** | **Animals** | **Changes in the composition of bacterial flora** | **Changes in abundance** | **Changes in metabolism** | **Intestinal barrier** | **Intestinal immunity** | **Health** | **Reference** |
| --- | --- | --- | --- | --- | --- | --- | --- | --- | --- |
| essential oils (400 mg/kg) | carvacrol, thymol, and cinnamaldehyde | piglets |  | bacteria involving the Erysipelotrichaceae family, Holdemanella genus, Phascolarctobacterium genus, and Vibrio genus were enriched |  | significantly promoted jejunal goblet cells in the villus, jejunal mucosa ZO-1 mRNA expression, ileal villus height, and ileal villus height/crypt depth ratio |  | improves ADG and ADFI, improves intestinal epithelial development and intestinal immunity | (Shao et al., 2023) |
| 0.01% essential oil | 18% thymol and cinnamaldehyde | piglets | E. coli in the cecum, colon and rectum were reduced, the ratio of Lactobacilli to E. coli was increased in the colon, Total aerobe numbers in the rectum were decreased |  |  | Villus height to crypt depth ratio in the jejunum was greater |  |  | (Li et al., 2012a) |

**Table S1.** *Cont.*

| **Essential oil** | **Main components** | **Animals** | **Changes in the composition of bacterial flora** | **Changes in abundance** | **Changes in metabolism** | **Intestinal barrier** | **Intestinal immunity** | **Health** | **Reference** |
| --- | --- | --- | --- | --- | --- | --- | --- | --- | --- |
| Essential Oils | oregano extract：peppermint EO：  thyme EO = 99:0.3:0.7 | pigs | the bacterial amount of four genera was much higher in the ileum content, Escherichia (32.2%), Lactobacillus (27.4%), Clostridium (17.1%), and Terrisporobacter (9%); Prevotella copri (8.8%) and Barnesiella intestinihominis (8.4%) in the colon |  |  |  |  | increase of probiotic bacteria in jejunum, caecum and colon | (Ruzauskas et al., 2020) |
| *oregano* essential oil (0.02%, 0.04%, 0.08%) | the purity of oregano essential oil was 5% | rabbits |  |  |  | the Villus high/Crypt depth value in the ileum increased significantly, increased significantly the gene expression of junctional adhesion molecule 2 (JAM2) and JAM3 in jejunum | the content of interleukin-2 (IL-2) and immunoglobulin A (SIgA) in jejunum and IL-10 and secretory immunoglobulin G (SIgG) in ileum were significantly increased | modulates immune responses and enhances the intestinal barrier | (Li et al., 2021) |

**Table S1.** *Cont*

| **Essential oil** | **Main components** | **Animals** | **Changes in the composition of bacterial flora** | **Changes in abundance** | **Changes in metabolism** | **Intestinal barrier** | **Intestinal immunity** | **Health** | **Reference** |
| --- | --- | --- | --- | --- | --- | --- | --- | --- | --- |
| 25 mg/kg of *Oregano* essential oil |  | pigs | lower population of Escherichia coli in the jejunum, ileum, and colon |  |  | increased villus height and expression of occludin and zonula occludens-1 (ZO-1) in the jejunum |  | can reduce the production of proinflammatory cytokines and promote the integrity of the intestinal barrier | (Zou et al., 2016) |
| *Orange* essential oil |  | mice |  | significantly changed the structure of the flora and increased the diversity of the intestinal microflora, increased the abundance of Bacteroidetes and Lactobacillus in the intestine |  |  |  |  | (Qu et al., 2023) |
| *cinnamon bark* oil (CNO), *clove bud* oil (CLO) and *ajwain seed* oil (AJO) |  | broiler chickens | Escherichia coli in pre-caecal contents decreased |  |  | CNO significantly increased the villi height in duodenum, jejunum and ileuim |  | beneficial effects on immune response, gut health, antioxidant status and blood cholesterol | (Chowdhury et al., 2018) |

**Table S1.** *Cont*

| **Essential oil** | **Main components** | **Animals** | **Changes in the composition of bacterial flora** | **Changes in abundance** | **Changes in metabolism** | **Intestinal barrier** | **Intestinal immunity** | **Health** | **Reference** |
| --- | --- | --- | --- | --- | --- | --- | --- | --- | --- |
| plant EO blend | 5% carvacrol, 3% cinnamaldehyde, and 2% capsicum oleoresin | ducks |  | increased the abundances of short-chain fatty acid-producing bacteria (e.g., Subdoligranulum and Shuttleworthia) and decreased abundances of potential enteric pathogenic bacteria (e.g., Alistipes, Eisenbergiella, and Olsenella |  |  |  | improving antioxidant capacity, enhancing the intestinal barrier function and favorably modulating gut microbiota. | (Ge et al., 2023) |
| *oregano* essential oil and *Enviva* essential oil | (OEO:comprised of 5% thymol and 65% carvacrol) (EEO:4.5% cinnamaldehyde and 13.5% thymol) | ducks | decreased the cecal populations of Coliforms, total aerobes and lactose-negative Enterobacteria |  |  |  |  |  | (Abouelezz et al., 2019) |
| *oregano* essential oil | carvacrol and thymol were ≥5.5% and ≥0.15% | sheep |  | can improve the richness of intestinal flora and improve the intestinal flora structure. |  | in the ileum, duodenum, and jejunum, the V/C ratios were significantly higher |  | proving the production performance | (Sun et al., 2022) |

**Table S1.** *Cont*

| **Essential oil** | **Main components** | **Animals** | **Changes in the composition of bacterial flora** | **Changes in abundance** | **Changes in metabolism** | **Intestinal barrier** | **Intestinal immunity** | **Health** | **Reference** |
| --- | --- | --- | --- | --- | --- | --- | --- | --- | --- |
| *oregano* essential oil (0.25, 0.5, and 1 g/kg) |  | nile tilapia (fish) |  |  |  | branching of villi and increased villi length and width |  | increasing the growth performance, regulating the blood biochemical traits, improving the immune and antioxidative response | (Magouz et al., 2022) |
